# Supplementary material for: Proteomic remodeling during tumor cell-induced platelet aggregation unveils metastatic drivers in colorectal cancer
Source: Cancer Cell Int. 2026 Mar 27;26:179. doi: 10.1186/s12935-026-04282-7 (PMC13123001; doi:10.1186/s12935-026-04282-7)
Supplement: Supplementary file 2 — Supplementary Material 2 [file 12935_2026_4282_MOESM2_ESM.docx]

Proteomic Remodeling During Tumor Cell-Induced Platelet Aggregation Unveils Metastatic Drivers in Colorectal Cancer

T. Sauer et al.

**Additional File 2**

**
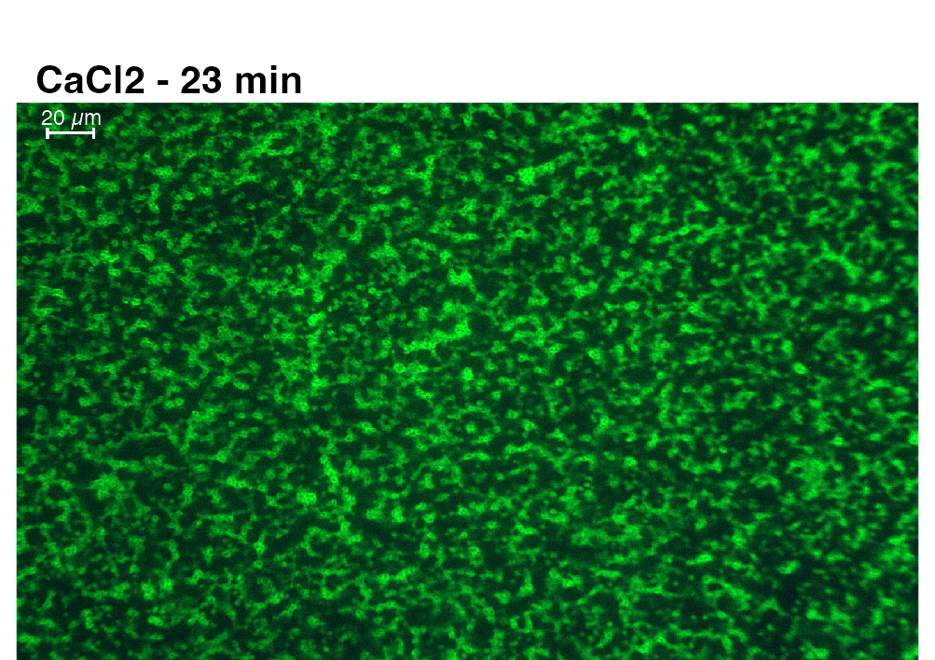
**

**Supplementary Figure 1:** Immunofluorescence microscopy of CaCl_2_-exposed platelets. Platelets labeled with anti-CD42b antibody (green) after 23 minutes of CaCl_2_ exposure. No visible platelet aggregation is observed, serving as a negative control for TCIPA experiments.

**
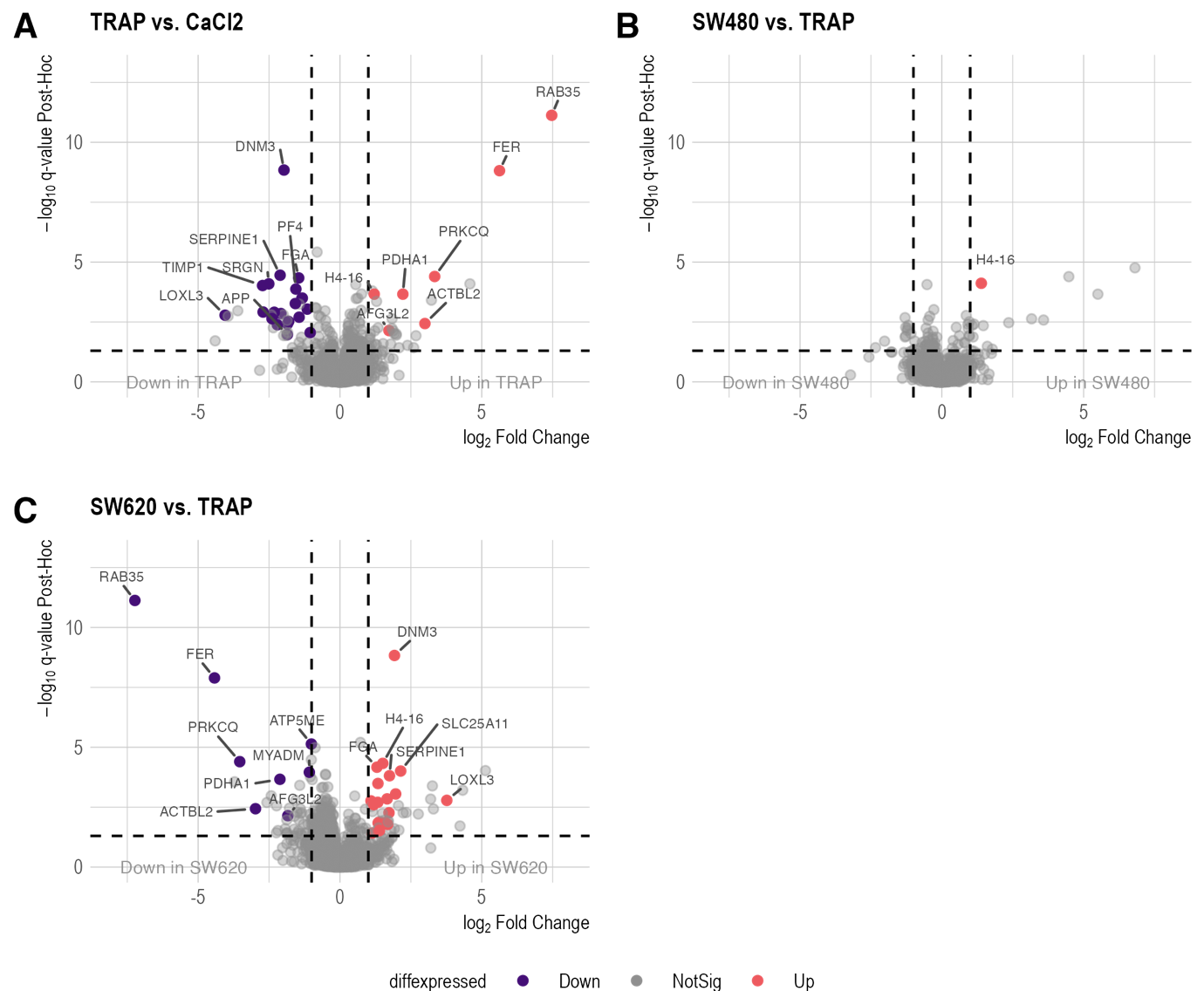
**

**Supplementary Figure 2:** Additional volcano plots of platelet sediment proteomics. A-D: Volcano plots illustrating differential expression in platelet sediments for three comparisons: A: TRAP *versus* CaCl2, B: SW480 *versus* TRAP and C: SW620 *versus* TRAP. Proteins are considered significantly abundant at ANOVA q-value and post-hoc q ≤ 0.05 and |log_2_FC| of ≥1. Significant proteins are highlighted in red, while downregulated proteins are shown in purple.

**
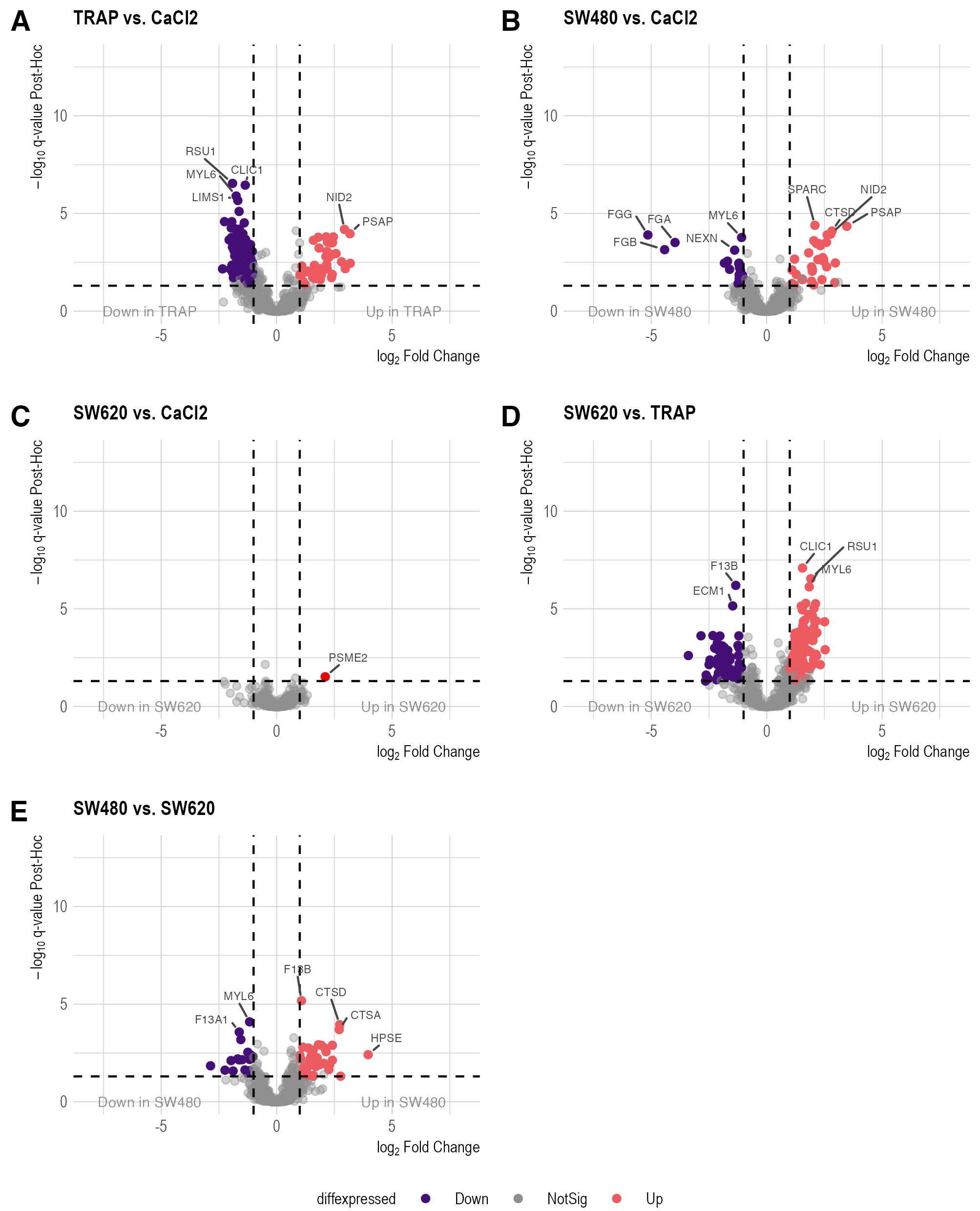
**

**Supplementary Figure 3:** Additional volcano plots of platelet secretome proteomics. A-E: Volcano plots illustrating differential expression in platelet sediments for three comparisons: A: TRAP *versus* CaCl2, B: SW480 *versus* CaCl_2_, C: SW620 *versus* CaCl_2_, D: SW620 *versus* TRAP, and E: SW480 *versus* SW620. Proteins are considered significantly abundant at ANOVA q-value and post-hoc q ≤ 0.05 and |log_2_FC| of ≥1. Significant proteins are highlighted in red, while downregulated proteins are shown in purple.

**Supplementary Figure 4:**

**
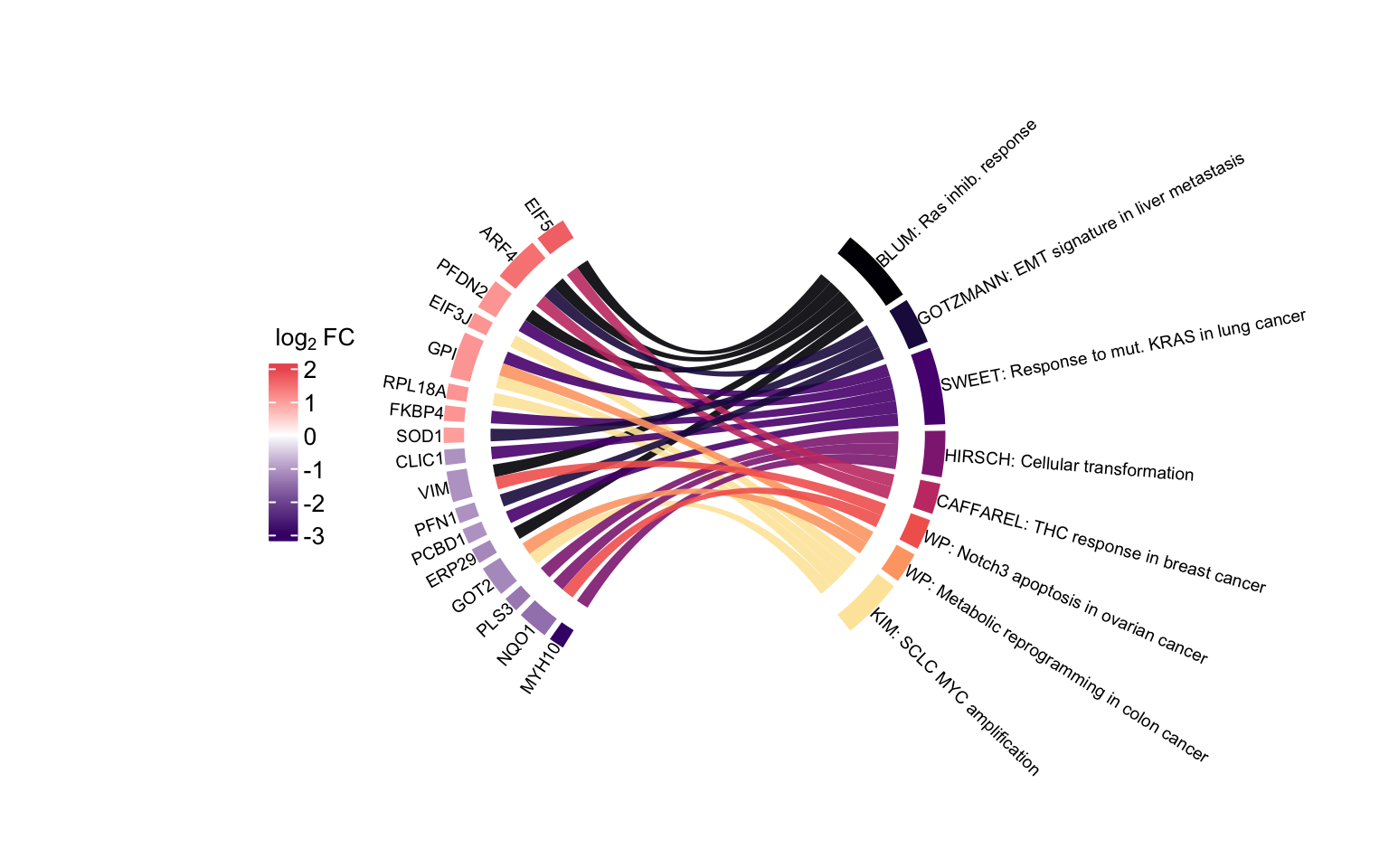
**

**Supplementary Figure 4:** Functional analysis of cancer and metastasis-associated genes. Chord diagram illustrating the results of an over-representation analysis using the MsigDB C2 collection. The diagram maps significantly enriched gene sets (q-value ≤ 0.05) containing ‘cancer’ and/or ‘metastasis’ terms to 17 cancer and metastasis-associated genes. Gene nodes are color-coded based on their log2FC between platelet-exposed SW480 and SW620 cells. Gene set labels display the author of the source publication (except for WP, denoting WikiPathways) and abbreviated gene set names for clarity.
